# A Cross-Sectional Study of Large Language Models in Lung Cancer Information Delivery: Readability, Quality, and Patient-Centred Evaluation

**DOI:** 10.3390/healthcare14121769

**Published:** 2026-06-18

**Authors:** Ömer Önal, Suzan Temiz Bekce

**Affiliations:** 1 Department of Thoracic Surgery, Faculty of Medicine, Erciyes University, Kayseri 38039, Turkey; omeronal@erciyes.edu.tr; 2 Department of Thoracic Surgery, Kayseri City Hospital, Kayseri 38080, Turkey

**Keywords:** lung cancer, large language models, patient education, health literacy, health promotion, vulnerable populations, health promotion programmes, readability, artificial intelligence

## Abstract

**Highlights:**

**What are the main findings?**

- ChatGPT-4o mini demonstrated superior readability (~6th-grade level), significantly lowering the cognitive barrier for vulnerable patient populations compared to Gemini and Copilot.
- No hallucinations were observed across all March 2026 model versions, indicating a major evolution in Artificial Intelligence (AI) reasoning and reliability since early 2023–2024 benchmarks.

**What are the implications of the main findings?**

- LLMs facilitate a paradigm shift from static, “one-size-fits-all” brochures to tailored, query-specific information delivery, enabling highly personalized patient education in oncology.
- These models should be integrated into health promotion programmes as supportive navigation tools that empower patients while remaining complementary to professional physician-led consultation.

**Abstract:**

**Background/Objectives**: Lung cancer is a leading cause of cancer-related mortality worldwide. As patients increasingly utilize large language models (LLMs) for health information, evaluating the readability and patient-centeredness of these tools is critical. This study aims to compare the performance of ChatGPT-4o mini, Microsoft Copilot, and Google Gemini in providing lung cancer information, focusing on their utility for individuals with limited health literacy. **Methods**: In this cross-sectional study (March 2026), 30 responses to ten standardized lung cancer-related queries were analyzed. Outputs were assessed using JAMA benchmarks and mDISCERN for quality, the SMOG index for readability, and PEMAT-P for understandability and actionability. Inter-rater reliability was analyzed using intraclass correlation coefficients (ICCs). **Results**: ChatGPT-4o mini demonstrated superior readability, achieving a sixth-grade level (SMOG: 6.23 ± 0.72, *p* < 0.001). Gemini achieved higher JAMA scores, indicating stronger academic rigour. While PEMAT-P scores were highest for ChatGPT (63.7%), all models exhibited moderate mDISCERN quality. Inter-rater reliability was excellent for JAMA (ICC = 1.000) and PEMAT-P (ICC = 0.883), though moderate for mDISCERN (ICC = 0.365), reflecting inherent interpretative subjectivity in qualitative assessment. No hallucinations were observed. **Conclusions**: Current LLMs exhibit a trade-off between accessibility and academic rigour: ChatGPT favours patient-friendly readability, while Gemini emphasizes structured content. The observed inter-rater variability in mDISCERN underscores the complexity of standardizing qualitative AI evaluation. These findings suggest that LLMs function best as complementary aids rather than substitutes for physician-led communication.

## 1. Introduction

Lung cancer remains one of the leading causes of cancer-related mortality worldwide and continues to represent a highly invasive and prevalent disease [1]. Given its aggressive nature, early diagnosis and appropriate treatment are of critical importance [2,3]. The tumour–node–metastasis (TNM) classification system, which evaluates primary tumour extent (T), lymph node involvement (N), and distant metastasis (M), plays a central role in disease staging and in guiding treatment decisions [4].

The clinical challenges present at the time of diagnosis, together with the subsequent treatment process, can create a highly complex and demanding situation for patients, thereby complicating overall clinical management. Although healthcare professionals remain the primary source of information, patients often seek additional information to evaluate treatment options and to cope with uncertainties. Given that the disease is associated with substantial morbidity and mortality, access to accurate and reliable medical information is of critical importance.

Large language models (LLMs) have increasingly emerged as widely used tools for accessing medical information [5,6,7,8], and cancer remains among the most frequently queried topics in these models [9]. Populations with lower health literacy have been reported to be at greater risk of exposure to misinformation generated by artificial intelligence [10]. Health literacy is a critical determinant of clinical outcomes in oncology. For patients with limited health literacy—often considered a vulnerable population—the complexity of lung cancer terminology can lead to significant anxiety and poor treatment adherence. However, significant concerns remain regarding the accuracy, reliability, and particularly the patient-level comprehensibility of information produced by these systems; such concerns are further amplified in conditions like lung cancer, where public awareness remains limited [11].

Studies published in 2023 have questioned the accuracy and consistency of responses provided by models such as ChatGPT and Google Bard in the context of lung cancer screening and terminology [12,13]. Following these early assessments, more recent evaluations conducted in 2025 indicated a trend toward enhanced performance consistency as models evolved [14]. Nevertheless, as LLMs transition from being static information repositories to dynamic interactive agents, their potential to deliver tailored, query-specific information that remains accessible to vulnerable populations while minimizing the risk of misinformation remains insufficiently explored.

In this study, the performance of ChatGPT, Gemini, and Microsoft Copilot was evaluated using ten standardized questions derived from common patient queries identified in the literature. The aim was to provide a multidimensional comparison and to critically evaluate the methodological constraints of using current assessment tools for dynamic AI outputs encompassing academic quality and transparency (benchmarks of the Journal of the American Medical Association [JAMA]), clinical reliability (Modified DISCERN [mDISCERN] instrument), readability (Simple Measure of Gobbledygook [SMOG] index), and patient-centred comprehensibility and actionability (Patient Education Materials Assessment Tool for Printable Materials [PEMAT-P]).

It is important to note that this study does not aim to validate LLMs for clinical decision-making, but rather to identify the current boundaries and performance characteristics of these systems as adjunct informational tools in oncology.

## 2. Materials and Methods

This cross-sectional study was conducted on 23 March 2026 to evaluate the quality, reliability, readability, and comprehensibility of lung cancer–related content generated by three widely used large language models (LLMs): ChatGPT (OpenAI, GPT-4o mini, San Francisco, CA, USA), Gemini (Google, Gemini 1.5 Flash, Google LLC, Mountain View, CA, USA), and Copilot (Microsoft, Redmond, WA, USA, free version, https://copilot.microsoft.com).

The set of ten standardized questions used in this study was developed based on similar studies in the literature and the most frequently reported concerns among patient populations [12,13,14]. The questions and their scope are presented in Table 1.

To eliminate any potential contextual bias from prior interactions, a completely new and independent chat session was initiated for each query across all models. Default model settings were used, and all AI-generated responses were subsequently anonymized to ensure an objective, brand-blinded evaluation.

The generated responses were evaluated independently by two board-certified thoracic surgeons. Quality and reliability assessments were performed using the mDISCERN and JAMA criteria. Readability was quantified using the SMOG index, and the PEMAT-P (Version 1.0) tool was used to evaluate understandability and actionability. To ensure assessment consistency and mitigate inter-rater variability, evaluators underwent a preliminary calibration session using three pilot samples to reach consensus on scoring criteria prior to the formal evaluation.

Statistical analyses were performed using Turcosa Analytics (Version 1.0) [15]. Data were presented as mean ± standard deviation (SD). The normality of distribution was assessed using the Shapiro–Wilk test. Given the ordinal nature of the scoring systems and the clustered data structure—where each of the 10 questions served as a consistent analytical unit across all three LLMs—we employed the Kruskal–Wallis test followed by Dunn’s post hoc test for pairwise comparisons. This approach provides greater robustness for datasets with limited sample sizes and non-normal distributions compared to parametric methods. Inter-rater reliability was assessed by calculating the intraclass correlation coefficient (ICC) using a two-way mixed-effects model. A *p*-value of <0.05 was considered statistically significant.

## 3. Results

A total of 30 responses (10 per model) generated by three LLMs were analyzed. mDISCERN scores did not differ significantly among ChatGPT (3.1 ± 0.21), Gemini (3.0 ± 0.20), and Copilot (2.9 ± 0.30) (H = 0.638, *p* = 0.727) (Table 2).

In contrast, significant differences were observed in JAMA benchmark scores (H = 11.23, *p* = 0.003). Gemini (1.7 ± 0.48) demonstrated significantly higher JAMA scores compared to ChatGPT and Copilot. Pairwise comparisons showed that Gemini scored significantly higher than the other models, whereas no significant difference was observed between ChatGPT and Copilot (Table 2).

The SMOG Index showed a statistically significant difference among models (H = 16.52, *p* < 0.001). ChatGPT demonstrated the lowest readability burden (6.23 ± 0.72), corresponding to an approximate 6-year education level. Both Copilot (9.84 ± 1.53) and Gemini (8.61 ± 1.67) demonstrated significantly higher SMOG scores, with no statistically significant difference between these two models (Table 2).

Total PEMAT-P scores differed significantly among models (H = 8.42, *p* = 0.0014). ChatGPT achieved the highest score (63.7%), followed by Gemini (59.1%) and Copilot (52.1%). Dunn’s post hoc analysis revealed a significant difference between ChatGPT and the other two models, whereas no significant difference was observed between Gemini and Copilot (Table 2).

Inter-rater reliability between the two thoracic surgeons is presented in Table 3. Excellent agreement was observed for JAMA scores (ICC = 1.000, *p* < 0.001) and PEMAT-P scores (ICC = 0.883, *p* < 0.001), whereas fair agreement was observed for mDISCERN scores (ICC = 0.365, *p* = 0.022).

## 4. Discussion

This study is among the limited number of investigations that evaluate large language model (LLM) outputs in lung cancer not only in terms of accuracy but also by jointly assessing patient-centred understandability and applicability. It has been reported that LLMs are increasingly being utilized in healthcare decision-making processes and medical text analysis [16,17,18].

With the advancement of technology, many individuals today increasingly use the internet alongside face-to-face consultations with healthcare professionals. The reasons for turning to internet-based sources include seeking information about clinical conditions, treatment options, and medications [19,20,21]. Zhong et al. highlighted in their systematic analysis that the interpretability of LLM outputs should be carefully evaluated [18].

A study conducted in 2024 suggested that although ChatGPT’s responses on lung cancer demonstrated a high level of readability, they may increase the risk of misinterpretation or, in some cases, may not be adequately understood [22]. In contrast, in the present study, evaluation using the SMOG index showed that ChatGPT generated more accessible content at approximately a sixth-grade reading level compared to Gemini and Copilot. This may indicate that ChatGPT provided content that appeared significantly more accessible for individuals with lower health literacy, potentially reducing the cognitive barrier for vulnerable patient populations compared to Gemini and Copilot, which required a higher level of education. Furthermore, PEMAT-P scores may indicate that ChatGPT provided higher levels of understandability than the other models. These findings may suggest that such outputs might have the potential to facilitate access to information, particularly among individuals with limited health literacy, although their impact on disease understanding and healthcare-seeking behaviour requires further clinical validation.

In a study conducted by Rahsepar et al. in 2023 [12], responses generated by ChatGPT and Google Bard (the earlier version of Gemini) to questions related to lung cancer prevention, screening, and commonly used radiological terminology were classified as correct, partially correct, incorrect, or unanswered in terms of accuracy. ChatGPT achieved the highest proportion of correct responses (70.8%), whereas Google Bard demonstrated a lower accuracy rate (51.7%). In addition, Bard was reported to refrain from answering certain questions and, in some cases, to generate hallucinated responses [12].

In contrast, in the present study, none of the evaluated models left any questions unanswered, and no clearly identifiable hallucinated responses were observed. This discrepancy may be partly explained by methodological differences. In particular, the use of standardized prompts in our study may have contributed to more consistent model behaviour. Furthermore, conducting all queries on a single day may have minimized variability related to temporal fluctuations in model performance. These factors may have reduced output instability and contributed to the absence of observable misleading responses. However, this controlled approach may not fully reflect real-world usage conditions, where model outputs can vary over time and across different interactions.

Following the initial studies in 2023, Nguyen et al. (2025) re-evaluated different LLMs using the same set of questions and reported that the GPT-o3-mini model achieved the highest accuracy rate, followed closely by Gemini [13]. However, these studies primarily focused on accuracy and did not evaluate readability or comprehensibility. In contrast, our study demonstrated high inter-rater agreement, supporting the reliability of the findings.

In another study conducted in 2023, questions about lung cancer and its screening were posed to LLMs both in a free-response format and with instructions to provide answers at a sixth-grade reading level. In that study, ChatGPT, GPT-4, and Bard models were used; readability was assessed using the Flesch Reading Ease index, and accuracy was evaluated by three experts. The findings demonstrated that simplified responses improved readability across all three models. However, the overall readability level was still reported to be challenging for the average adult patient, remaining above the eighth-grade level [14].

Similarly, in our study, ChatGPT demonstrated a readability level of approximately a sixth-grade level based on SMOG scores, whereas Gemini and Copilot required higher reading levels, with ChatGPT demonstrating significantly lower SMOG scores, suggesting higher readability. Moreover, despite the absence of additional prompting (such as instructions for simplification), the overall comprehensibility of the responses suggests an improvement in the ability of LLMs to generate user-centred content over time.

When assessed using the mDISCERN tool, the textual quality of all models was found to be broadly similar. In contrast, the JAMA benchmark, which evaluates academic quality and source attribution, showed that Gemini achieved significantly higher scores. These findings suggest a potential trade-off: while ChatGPT provides more accessible, patient-friendly information, Gemini appears to prioritize structured, reference-based responses. The observation that all models achieved moderate mDISCERN scores suggests that LLMs may still have limitations in presenting medical information.

These findings suggest a trade-off between readability and academic rigour in LLM-generated content. While ChatGPT provides more accessible and patient-friendly information, Gemini appears to prioritize structured, reference-based responses, which may be associated with reduced readability.

A notable methodological point in our study is the moderate inter-rater reliability observed for mDISCERN scores (ICC = 0.365). While this value suggests a degree of evaluator variability, it aligns with recent findings in the literature, where similar qualitative assessments of AI-generated content have reported moderate agreement levels (e.g., κ = 0.55) as sufficient for reproducible scoring [23]. Furthermore, this result highlights the interpretative complexity inherent in traditional medical communication tools when applied to non-static AI-generated responses, a challenge recently reinforced by comparative analyses [24].

In contrast, our study demonstrated excellent inter-rater agreement for more structured instruments like JAMA (ICC = 1.000) and PEMAT-P (ICC = 0.883), which suggests that when assessment criteria are objective and structured, high consistency is achievable even with LLM-generated content. This discrepancy illustrates a critical methodological insight: while AI-generated medical information can be reliably assessed for academic structure and patient-friendly readability, ‘clinical quality’ remains inherently dependent on the evaluator’s interpretation. Consequently, our mDISCERN findings should be interpreted with this inherent subjectivity in mind, reflecting the ongoing challenge of standardizing qualitative evaluations for dynamic AI outputs.

In terms of comprehensibility and applicability, the total PEMAT-P assessment demonstrated that ChatGPT appeared to achieve significantly higher scores compared to the other models. This finding may suggest that ChatGPT might have greater potential for generating more patient-friendly educational content. While ChatGPT appeared to perform better in terms of accessibility and Gemini in terms of academic rigour, these tools should currently be considered complementary to, rather than replacements for, the patient–surgeon dialogue in lung cancer management.

## 5. Conclusions

LLMs demonstrate potential as supportive tools for patients seeking information about lung cancer. Among the evaluated models, ChatGPT demonstrated advantages in terms of readability and patient comprehension, whereas Gemini showed stronger performance in academic content quality and source utilization. Our findings underscore that while AI can bridge the gap in health literacy, it must be carefully integrated into health promotion programmes to ensure accuracy alongside simplicity. However, the overall moderate quality of content generated by all models represents an important limitation. Although the responses were largely comprehensible and hallucination rates appeared to be low, limitations in source transparency and the lack of clinical context indicate that these systems are not yet suitable as a replacement for professional medical advice.

LLMs have considerable potential as supportive tools that enhance patient autonomy. Future research should focus on optimizing artificial intelligence not merely as an “encyclopaedia” for information provision, but also as a “navigation system” that improves health literacy and supports physician–patient communication. Such an approach may reduce cognitive load and facilitate clearer, more structured access to information during complex treatment processes.

In clinical practice, LLMs may help reduce uncertainty during consultations by providing patients with preliminary information regarding symptoms, diagnostic pathways, and disease-related terminology. In addition, these systems may function as post-visit supportive tools by simplifying and reinforcing information provided by physicians, thereby improving patient recall, understanding, and treatment adherence. Nevertheless, given that clinical decision-making responsibility remains with healthcare professionals, these tools should be regarded as complementary rather than substitutive.

Future studies should focus on several specific areas to enhance the clinical applicability of LLMs in oncology education. First, the consistency of model performance across different languages should be evaluated. Second, real-world studies involving individuals with varying levels of health literacy are needed. Finally, interventional studies are required to investigate whether structured integration of LLMs can improve measurable patient outcomes. In conclusion, while ChatGPT appears to perform better in terms of accessibility and Gemini in terms of academic rigour, these tools should currently be considered as complementary aids that support, rather than replace, the patient–surgeon dialogue in lung cancer management.

### Limitations

The findings of this study are based on a limited set of questions related to lung cancer, and the results may not be generalizable to other cancer types. Furthermore, as our evaluations were conducted exclusively in English, performance metrics may vary across different linguistic contexts; therefore, validation of these findings in diverse clinical and linguistic environments is warranted.

This study has several limitations that warrant consideration. First, the absence of an expert-generated ‘gold standard’ reference reflects our intentional research design. By prioritizing the assessment of patient-centred cognitive accessibility and utility, our study provides a distinct perspective on how LLMs bridge the communication gap between complex oncology data and layperson understanding. This focus serves as a foundational assessment of the communicative performance of LLMs in oncology education, complementing existing clinical accuracy audits.

Second, we acknowledge that the involvement of two thoracic surgeons as evaluators carries inherent limitations regarding generalisability. To mitigate this and ensure assessment robustness, we implemented a pre-evaluation calibration session and pilot scoring to align our scoring criteria before the formal evaluation.

Regarding the evaluation methodology, we acknowledge that tools such as mDISCERN and PEMAT-P were originally designed for static educational materials. The inherent interpretative subjectivity of mDISCERN likely contributed to lower inter-rater reliability compared to more structured instruments like JAMA and PEMAT-P. However, this discrepancy highlights a critical methodological insight: as AI-generated medical information becomes more prevalent, there is an urgent need to adapt or develop assessment instruments specifically calibrated for the dynamic nature of AI-generated text.

Finally, regarding the statistical approach, while our analysis was performed at the question level using clustered data, we employed the Kruskal–Wallis test to provide a clear benchmark for model-specific performance as discrete units.

## Figures and Tables

**Table 1:** List of questions presented to large language models.

No	Category	Query Text
1	Pathophysiology	What exactly is lung cancer and how is it eradicated from the body?
2	Prognosis	Is this disease always fatal, or is there a chance of recovery?
3	Symptomatology	What are the earliest signs of lung cancer?
4	Triage	What symptoms should prompt me to see a doctor immediately?
5	Differential Diagnosis	Is a cough alone considered a symptom?
6	Epidemiology	Can someone who has never smoked still get lung cancer?
7	Etiology and Prevention	Besides smoking, what causes this disease, and what can we do to protect ourselves?
8	Diagnostic Methods	What tests (CT * scan, biopsy, etc.) are performed on a patient suspected of having lung cancer?
9	Staging	What do the stages of lung cancer mean?
10	Treatment	How is lung cancer treated?

* CT: Computed Tomography.

**Table 2:** Comparative analysis of LLM performance in lung cancer patient education.

Assessment Category	ChatGPT (n = 10)	Gemini (n = 10)	Copilot (n = 10)	H-Statistic	*p*-Value
Reliability					
mDISCERN (Score)	3.1 ± 0.21	3.0 ± 0.20	2.9 ± 0.3	0.638	0.727
Academic Quality					
JAMA (Score)	1 ± 0.00 ^a^	1.7 ± 0.48 ^b^	1.1 ± 0.31 ^a^	11.23	0.003
Readability					
SMOG Index	6.23 ± 0.72 ^a^	8.61 ± 1.67 ^b^	9.84 ± 1.53 ^b^	16.52	<0.001
Comprehension					
PEMAT-P Total (%)	63.7 ± 9.6 ^a^	59.1 ± 4.4 ^b^	52.1 ± 3.7 ^b^	8.42	0.0014

Values are expressed as mean ± standard deviation (SD). *p*-values were determined using the Kruskal–Wallis test, followed by Dunn’s post hoc test for pairwise comparisons. Different superscript letters (a, b) within the same row indicate statistically significant differences (*p* < 0.05). JAMA: *Journal of the American Medical Association*; mDISCERN: Modified DISCERN; SMOG: Simple Measure of Gobbledygook; PEMAT-P: Patient Education Materials Assessment Tool for Printable Materials.

**Table 3:** Inter-rater reliability (ICC) for mDISCERN, JAMA, and PEMAT-P scores.

Scale	Sample (n)	ICC	95% Confidence Interval	*p* Value
mDISCERN	30	0.365	0.073–0.600	0.022
JAMA	30	1.000	1.000–1.000	<0.001
PEMAT P (Total)	30	0.883	0.792–0.935	<0.001

ICC, Intraclass Correlation Coefficient; CI, Confidence Interval. A two-way mixed-effects model (absolute agreement) was used to calculate ICC.

## Data Availability

The data presented in this study are available on request from the corresponding author. The data are not publicly available due to the volume of AI-generated responses analyzed.

## Abbreviations

The following abbreviations are used in this manuscript:

AI	Artificial Intelligence
CT	Computed Tomography
ICC	Intraclass Correlation Coefficient
JAMA	Journal of the American Medical Association
LLM	Large Language Model
mDISCERN	Modified DISCERN (quality criteria for consumer health information)
PEMAT-P	Patient Education Materials Assessment Tool for Printable Materials
SMOG	Simple Measure of Gobbledygook (readability index)
TNM	Tumour–Node–Metastasis (staging system for cancer)
